# Treating to target of minimal disease activity and normal function in polyarticular juvenile idiopathic arthritis with adalimumab: analysis from a phase 3 clinical trial

**DOI:** 10.1186/1546-0096-12-S1-P9

**Published:** 2014-09-17

**Authors:** Nicola Ruperto, Daniel Lovell, Pierre Quartier, Angelo Ravelli, Mahinda Karunaratne, Jasmina Kalabic, Anabela Cardoso, Alberto Martini, Gerd Horneff

**Affiliations:** 1 PRINTO-IRCCS, Genova, Italy; 2 PRCSG, Cincinnati Children's Hospital Medical Center, Cincinnati, USA; 3 Hopital Necker-Enfants Malades, Paris, France; 4 Istituto G. Gaslini and Università degli Studi di Genova, Genova, Italy; 5 AbbVie Inc., North Chicago, USA; 6 AbbVie Deutschland GmbH & Co. KG, Ludwigshafen, Germany; 7 AbbVie, Amadora, Portugal; 8 Asklepios Klinik Sankt Augustin, Sankt Augustin, Germany

## Introduction

The Juvenile Arthritis Disease Activity Score (JADAS) ^1^ is becoming widely accepted in juvenile idiopathic arthritis (JIA) for defining a treat to target strategy.

## Objectives

To evaluate patients (pts) treated with adalimumab (ADA) (±methotrexate [MTX]) that achieved minimal disease activity (MDA) and both MDA and normalization of function.

## Methods

This *post hoc* analysis assessed pts aged 4-17 with polyarticular JIA enrolled in a phase 3 clinical trial (DE038)^2^, which consisted of a 16 week (wk) open-label (OL) lead-in with ADA±MTX, 32wk double-blind (DB) phase with ADA or placebo (PBO)±MTX, and OL extension (OLE) with ADA±MTX up to 346wks. Outcomes were assessed by 27-joint JADAS (JADAS27), based on C-reactive protein, and Childhood Health Assessment Questionnaire Disability Index (CHAQ-DI). MDA was defined as JADAS27<3.8 and normal function as CHAQ-DI<0.5. Pts who entered the DB phase were included; data were stratified by MTX treatment (tx) at entry.

## Results

At baseline, 75 pts on MTX had a mean JADAS27 of 21.2 and CHAQ-DI of 0.9, and 58 pts who were MTX naïve or had withdrawn from MTX had a mean JADAS27 of 23.8 and CHAQ-DI of 1.2. After 16wks of OL ADA, the mean JADAS27 was 6.1 and 6.7 and CHAQ-DI was 0.4 and 0.5 for ADA+MTX and ADA-MTX, respectively. Clinical improvements were seen at wk48 and wk88, and the mean JADAS27 at wk88 was 2.6, 3.0, 4.3, and 5.0 for ADA+MTX, ADA-MTX, PBO+MTX, and PBO-MTX, respectively. No pts had MDA or normal function at baseline; however, a good proportion achieved MDA and normal function during OL ADA. Fewer pts achieved MDA and normal function in the PBO tx compared with ADA continuation at both wk48 and wk88. Table 1.

**Table 1:** 

n (%)	Minimal Disease Activity				
	
	ADA Continuation		PBO		P-value
	
	+MTX	-MTX	+MTX	-MTX	
Week 16	28 (37.3)	19 (33.9)	NA	NA	NA

Week 48	19 (76.0)	14 (56.6)	15 (62.5)	13 (68.4)	0.8919

Week 88	26 (83.9)	20 (76.9)	14 (50.0)	13 (65.0)	0.0075

	Minimal Disease Activity with Normal Function				

Week 16	21 (28.0)	15 (26.8)	NA	NA	NA

Week 48	17 (68.0)	12 (48.0)	15 (62.5)	13 (68.4)	0.5155

Week 88	24 (77.4)	17 (65.4)	14 (50.0)	11 (55.0)	0.0362

P-value based on Cochran-Mantel-Haenszel statistics to test if there was a difference between ADA continuation vs. PBO.

## Conclusion

ADA±MTX resulted in a high percentage of pts achieving/sustaining MDA and normal function. Some improvement was seen with PBO during the DB period, but continued ADA tx shows better overall outcomes. A target of comprehensive disease control with MDA and normal function is achievable and aligned with current goals of JIA tx.

## Trial registration identifying number

NCT00048542

## Disclosure of interest

N. Ruperto Grant / Research Support from: AbbVie, AstraZeneca, BMS, Janssen Biologics BV, Eli Lilly & Co, "Francesco Angelini", GlaxoSmithKline, Italfarmaco, Novartis, Pfizer, Roche, Sanofi Aventis, Schwarz Biosciences GmbH, Xoma, Wyeth Pharmaceuticals, Employee of: GASLINI Hospital, Speaker Bureau of: Astellas, AstraZeneca, BMS, Italfarmaco, Janssen Biologics B.V., MedImmune, Roche, Wyeth/Pfizer, D. Lovell Consultant for: AbbVie, AstraZeneca, Centocor, BMS, Pfizer, Regeneron, Hoffman La-Roche, Novartis, UBC, Genentech, Xoma, Amgen, Forest Research, Speaker Bureau of: Wyeth Pharmaceuticals, P. Quartier Grant / Research Support from: AbbVie, Novartis, Pfizer, BMS, Chugai-Roche, Medimmune, Servier, Swedish Orphan Biovitrum, Consultant for: AbbVie, Novartis, Pfizer, BMS, Chugai-Roche, Medimmune, Servier, Swedish Orphan Biovitrum, A. Ravelli Grant / Research Support from: Pfizer, Consultant for: Hoffman La-Roche, Speaker Bureau of: Hoffman La-Roche, Centocore, BMS, Pfizer, Novartis, AbbVie, M. Karunaratne Shareholder of: AbbVie, Employee of: AbbVie, J. Kalabic Shareholder of: AbbVie, Employee of: AbbVie, A. Cardoso Shareholder of: AbbVie, Employee of: AbbVie, A. Martini Grant / Research Support from: AbbVie, AstraZeneca, BMS, Janssen Biologics BV, Eli Lilly & Co, "Francesco Angelini", GlaxoSmithKline, Italfarmaco, Naovartis, Pfizer, Roche, Sanofi Aventis, Schwarz Biosciences GmbH, Xoma, Wyeth Pharmaceuticals, Employee of: GASLINI Hospital, Speaker Bureau of: Astellas, AstraZeneca, BMS, Italfarmaco, MedImmune, G. Horneff Grant / Research Support from: AbbVie, Pfizer, Roche, Speaker Bureau of: AbbVie, Novartis, Pfizer, Roche.
